# Impact of exercise with blood flow restriction on muscle hypertrophy and performance outcomes in men and women

**DOI:** 10.1371/journal.pone.0301164

**Published:** 2025-01-28

**Authors:** Dawson Nancekievill, Ken Seaman, Danielle R. Bouchard, Amy M. Thomson, Martin Sénéchal

**Affiliations:** 1 Cardiometabolic, Exercise, and Lifestyle Laboratory, University of New Brunswick, Fredericton, NB, Canada; 2 Faculty of Kinesiology, University of New Brunswick, Fredericton, NB, Canada; Università degli Studi di Milano: Universita degli Studi di Milano, ITALY

## Abstract

Blood flow restriction training (BFRT) has been previously studied as an alternative form of resistance training to gain lean mass and improve performance outcomes. However, in all exercise studies of BFRT, the proportion of female participants represents only 17–29% of all research participants. This highlights a strong underrepresentation of females and the need for more knowledge on the impact of BFRT and sex differences. The primary objective was to compare the impact of 6-week BFRT on lean mass, strength, and performance outcomes between males and females. A total of 38 adults [age, 25.3 ±  3.1 years; female, n = 19 (50%)] performed whole-body resistance training program with blood flow restriction three times per week. Exercises were performed at 30% of 1-repetition maximum (1-RM) and blood flow restriction cuffs were set to 60% of each individual’s limb occlusion pressure. Body composition was assessed via dual-energy x-ray absorptiometry and strength was measured using 1-RM. A significant increase in lean mass was observed in males (*p* = 0.009) and females (*p* = 0.023) with no difference in the change between groups (*p* = 0.279). Both males and females increased 1-RM for upper- and lower-body exercises, with significant interaction effects (time x sex) for chest press (*p* = 0.003), seated row (*p* = 0.038), knee flexion (p = 0.043), and knee extension (*p* = 0.035), suggesting males increased 1-RM more for these exercises. Furthermore, peak power was improved in males (*p* < 0.001) and females (*p* = 0.002) during a vertical squat jump, but a significant interaction (time x sex) effect was observed (*p* = 0.039), suggesting males increased to a greater extent. Males and females significantly increased lean body mass, to a similar degree, following six weeks of resistance training in combination with blood flow restriction. Likewise, both males and females improved muscle strength following 6-week BFRT, however males may improve strength to a greater extent than females.

## Introduction

Exercise, including resistance training, has been called a “medicine” as it prevents and reduces the risk for chronic conditions such as type 2 diabetes mellitus, cardiovascular disease, and cancer [1,2]. One possible explanation for this risk reduction is the alteration in body composition, characterized by increased skeletal muscle mass [3–5]. This increase in muscle mass, known as hypertrophy, occurs with moderate to heavy-load resistance training and resistance training to volitional failure [6,7]. However, blood flow restriction training (BFRT) has emerged as an alternative to heavy-load resistance training. BFRT involves the utilization of a pneumatic cuff placed around the proximal region of the exercising limb to partially reduce blood flow combined with very light loads [8].

Although the exact physiological mechanisms of hypertrophy following BFRT are not fully understood, there is substantial evidence that BFRT can induce muscle hypertrophy [9–12]. A BFRT study using low-load BFRT performed to failure saw an increase in thigh muscle thickness similar to the traditional resistance training group despite a 33% lower total exercise volume [13]. Similarly, several BFRT meta-analyses indicated that BFRT can increase shoulder lean mass, pectoralis major thickness, and that muscle hypertrophy was comparable to high-load resistance training [9,14]. Interestingly, these increases in muscle mass translate into improvement in performance outcomes. Following a 4-week whole-body BFRT intervention, significant 1-repetition maximum (1-RM) improvements were observed in healthy adults for the knee extension, back squat, calf raise, and seated row [15]. Furthermore, several systematic reviews and meta-analyses support the impact of BFRT on muscle strength [9,11,14,16,17].

Although these findings are interesting, there is a currently growing interest in physiological sex differences in exercise training, of which there are many [18,19]. For instance, females tend to have greater resistance to fatigue during exercise, but males tend to exhibit greater strength, especially in the upper body [18,20]. Also, females have been shown to undergo less contractile dysfunction during high-intensity exercise than males, possibly due to decreased metabolic stress [18]. In addition, females have higher relative proportions of Type I versus Type II muscle fibers than males. Finally, females have greater oxidative capacity than males, likely due to their greater relative proportion of Type I muscle fibers, and tend to undergo less metabolic stress during high-intensity exercise, which could translate to less adaptative stimulus than males at the same work intensity [18]. These established sex differences specifically reduced metabolic stress and increased fatigue-resistance (two potential mechanisms of BFRT adaptation) in females, laid the foundation for this study and our hypothesis of sex differences in lean body mass, strength, and performance outcomes.

Furthermore, even if clear physiological sex differences exist for typical exercise training, the vast majority of studies investigating BFRT include only college-aged males (i.e., between the ages of 18–25) [21]. In fact, a call to action from a BFRT review article highlights that in studies of chronic and acute BFRT, only 29% and 17%, of all participants were females [22]. This call is echoed in a more recent review highlighting the continued absence of young females in the literature [21]. This fact strengthens an urgent need to study the impact of BFRT in young females to understand muscle mass adaptation better and investigate if these changes translate into increased performance. Therefore, the primary objective of this study was to study sex-based differences and to compare the impact of 6-week whole-body BFRT on lean body mass, muscle strength, and performance outcomes in males and females.

## Methods

### Study design

**Fig 1:**
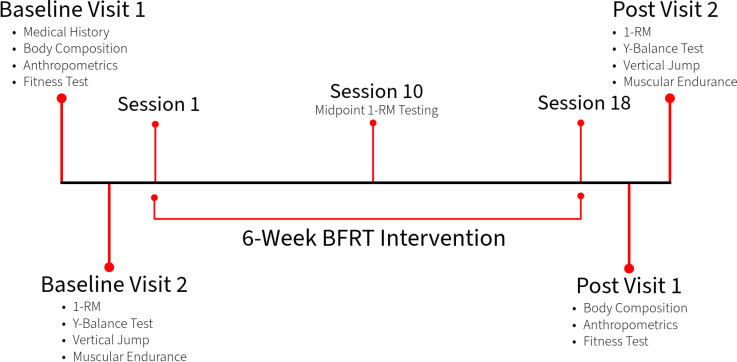
Study timeline.

The current project is a parallel control experimental study comparing men and women following a 6-week resistance training intervention in conjunction with partial blood-flow restriction. (Clinical Trial #: NCT05615831). Participants underwent baseline testing separated into two visits within a one-week span and presented at the Cardiometabolic Exercise and Lifestyle Laboratory at the University of New Brunswick for each visit. Participants began six weeks of BFRT within one week of the last baseline testing visit. Following the intervention, participants underwent follow-up testing at least two days [23], but no more than one week [10], following the last exercise session. An overview of the study design and timeline can be seen in Fig 1. All exercise sessions were supervised by research staff at the Cardiometabolic Exercise and Lifestyle Laboratory at the University of New Brunswick. Participants were told to wear comfortable clothes. Not every session was necessarily completed at the same time of day for all participants, as such, time of day was recorded for each exercise session and testing visit. All participants provided written and informed consent prior to participation. The project was reviewed and approved by the University of New Brunswick Research Ethics Board (REB 2021-124).

### Sample size

A power calculation was performed using G-power software (version 3.1.9.4, Germany) to determine the appropriate sample size for statistical significance. Based on an alpha of 0.05, a power of 0.8, and an effect size of 0.4, we determined the required total sample size to be eight participants for a repeated measures analysis of variance (ANOVA). However, we anticipate a dropout percentage of 13% as reported by Høgsholt et al. (2022) [24]. Furthermore, it has been suggested that in order to detect interaction effects between sexes, as well as main effects, the sample size needs to be four-times the size [25]. To account for dropout rate, and to ensure adequate ability to detect interaction and main effects, we attempted to recruit approximately 20 per group for a total of 40 participants.

### Participation

#### Inclusion criteria.

Participants were eligible for inclusion if they were between the ages of 19 and 30 years. In addition, participants had to be physically inactive, but otherwise healthy. Physical inactivity was defined as not meeting the World Health Organization’s 2020 physical activity guidelines: 150 minutes of moderate-vigorous physical activity and two muscle-strengthening activities per week [26]. Physical activity levels were estimated through questionnaires and using Fitbit Charge 3 activity trackers. It was shown in a 2018 systematic review that Fitbit activity trackers provide accurate measures of steps in adults with no mobility limitations [27]. Using a pre-determined threshold (10,000 steps/day) as the minimum number of steps required to reach moderate intensity physical activity, anybody who averaged under 10,000 steps/day over a 4–7 day window and did not perform muscle strengthening activities twice per week was considered physically inactive [28].

#### Exclusion criteria.

Exclusion criteria included: 1) aged outside prearranged threshold (19–30 years), 2) the presence of cardiovascular disease such as coronary heart disease, uncontrolled hypertension, peripheral vascular disease, venous thromboembolism, other blood clotting disorders, or hemophilia, 3) surgery, bone fracture, or a skin graft within the last three months, 4) pregnancy, and 5) meeting or exceeding physical activity guidelines.

#### Recruitment.

**Fig 2:**
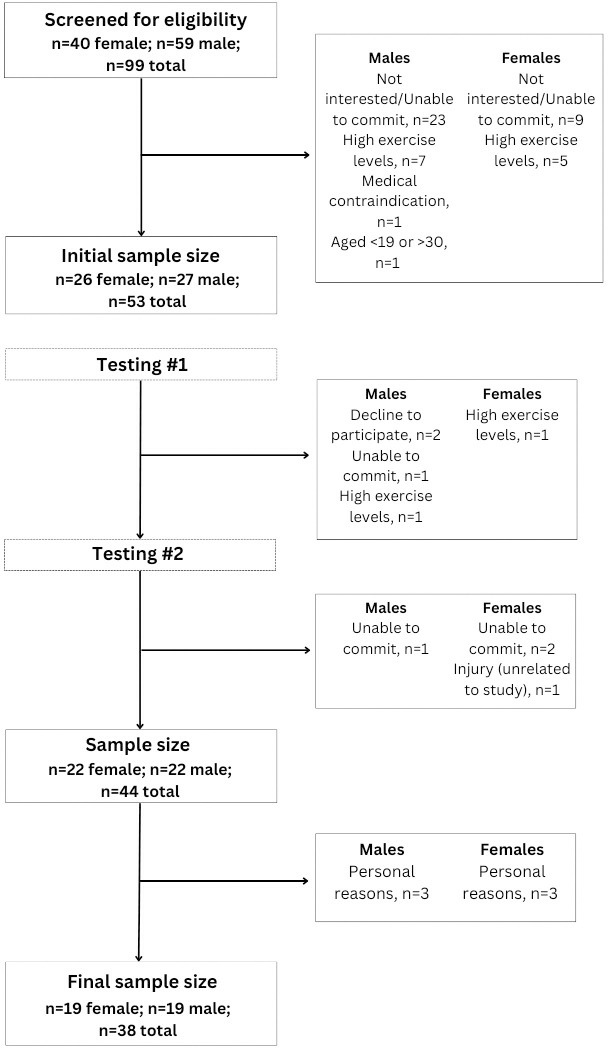
Participant flowchart.

Recruitment was performed between May 2022 and July 2023 through the distribution of promotional flyers, University of New Brunswick’s newsletter, and social media advertisements through Facebook and Instagram. An overview of recruitment can be seen in Fig 2.

### Exposure variable – blood flow restriction training

Participants undertook 6-weeks of whole-body resistance training in conjunction with blood flow restriction to their exercising limbs. The intervention consisted of three supervised exercise sessions per week consisting of five different exercises: knee flexion (hamstring curl), knee extension, leg press, chest press, and seated row. As the exercise load was quite low, no warmup procedure occurred prior to beginning the exercise session. The exercise load was individualized to 30% of each participant’s 1-RM for each exercise. Participants were required to complete 75 total repetitions broken into four sets for each exercise. The sets were broken up in the following manner: set 1: 30 repetitions; set 2: 15 repetitions; set 3: 15 repetitions; set 4: 15 repetitions, as this protocol has previously been used in blood flow restriction research to induce muscle hypertrophy in a variety of populations [29,30] and has been suggested by multiple reviews [31,32]. At week 4, participants had their 1-RM reassessed to adjust the 30% 1-RM exercising loads. This occurred during the first exercise session of week 4. As such, following the 1-RM reassessment, participants performed two sets per exercise (30 reps and 15 reps) using the newly adjusted 30% 1-RM weight, before returning to the original rep scheme for their next session.

Blood flow restriction cuffs were placed at the most proximal portion of the exercising limb (just above biceps brachii on the arm and near the inguinal crease on the thigh), which is what has previously been used in BFRT research [8,29–31,33–36]. Blood flow restriction was achieved using the KAATSU C3 device (KAATSU Global, Inc., Huntington Beach, CA, USA). The KAATSU arm and leg cuffs are 5 cm wide, respectively, and are single-bladder cuffs. Cuffs were inflated to 60% of each individual’s total limb occlusion pressure as this has been shown to be a safe and effective pressure to induce muscular adaptations [15], and is within the recommended pressure range for BFRT [8,31]. Each participant’s total limb occlusion pressure (LOP) was estimated using equations developed by Loenneke et al. (2015) listed here [33]:


$$Leg\;arterial\;occlusion\;\left(mmHg\right)=5.893\left(thigh\;circumference\right)+0.734\left(diastolic\;blood\;pressure\right)+0.912\left(systolic\;blood\;pressure\right)-220.046$$ (1)

$$Arm\;arterial\;occlusion\;\left(mmHg\right)=0.514\left(systolic\;blood\;pressure\right)+0.339\left(diastolic\;blood\;pressure\right)+1.461\left(arm\;circumference\right)+17.236$$ (2)

Although these equations have modest R-squared values of 0.61 and 0.49, respectively [33], they have been successfully utilized in a BFRT intervention of trained athletes leading to significant improvements in muscle hypertrophy and functional outcomes with no reported side effects [37]. Furthermore, although LOP was estimated, a study comparing the impact of various cuff pressures showed similar effects on muscle size, torque, strength, and endurance between 40% and 90% pressures [38]. These findings suggest that greater pressures are not necessarily more effective at inducing adaptations and that small pressure fluctuations won’t necessarily serve as greater or lesser stimuli during training. Cuffs remained inflated during the rest in between sets of each exercise but were deflated for the rest period between exercises [29,39–42]. The set rest was 60 seconds, and the rest between exercises was four minutes.

### Primary outcome measure – body composition differences between sexes

Lean body mass was estimated using dual-energy x-ray absorptiometry (DXA) prior to the 6-week BFRT intervention, and again following the intervention. Body composition was estimated using a Hologic Horizon^®^ DXA System (Hologic Canada ULC, Mississauga, ON, Canada). Lean body mass constitutes that which is not fat mass nor bone mineral mass [43]. Participants presented to the laboratory following a 12-hr fast and were asked to refrain from exercise for a 24-hr period prior to testing for consistency, and as dietary intake and exercise could alter lean mass [44]. Participants were instructed to wear loose-fitting clothing with no metal (buckles, zippers, buttons, etc.) and then instructed to lie supine on the scanner’s table and remain still for the duration of the scan. Arms were placed at the participants’ sides with palms facing medially and thumbs pointed upwards. For individuals too large for the width of the table, they were positioned with one arm outside of the scan area and results of the scanned arm were duplicated. The coefficient of variation in our lab for lean mass is 0.6% and for body fat percentage is 0.7%. This was performed on 33 people (males, n = 10) with a mean age of 23.4 years and a mean body mass index (BMI) of 25.6.

### Exploratory variables

Anthropometric measurements, muscular power and endurance, and strength were measured for exploratory purposes and sample description. Participants’ height and weight were measured to the nearest 0.5 cm and 0.1 kg, respectively, according to the CSEP protocol [45]. Weight was measured using a calibrated column scale (SECA^®^ model #213). Height was measured using a standardized stadiometer. With no shoes, feet together, and arms at their side, height was taken following an inhalation. Hip and waist circumference were measured using an anthropometric tape measure and recorded to the nearest 0.5 cm. For hip and waist measurements, participants stood with their feet shoulder-width apart and their arms folded across their chest. Waist circumference was measured at the upper lateral border of the iliac crest following a normal exhalation, hip circumference was measured around the widest portion of the buttocks after a normal exhalation [46].

Strength was assessed by 1-RM for the five exercises used during the intervention. 1-RM was measured during the second baseline testing visit, at the midpoint of the study during the first exercise session of week four, and again during the second testing visit in the follow-up testing. Each participant’s 1-RM was determined using the following protocol: one set of 6–10 repetitions, followed by one set of 3–5 repetitions, followed by small incremental increases for one repetition until a failure is achieved within seven attempts. If no failure was achieved within seven attempts, the 1-RM for that exercise was redone prior to their first exercise session.

Muscular power was estimated using the squat jump equation derived by Sayers et al. (1999) and is as follows [47]:


$$Peak\;Power\;\left(W\right)=60.7\;x\;\left(jump\;height\;\left[cm\right]\right)+45.3\;x\;\left(body\;mass\;\left[kg\right]\right)-2055$$ (3)

This equation was chosen as it has been shown to be more accurate than previously used power estimation equations and was developed from a large and diverse population which enhances our external validity [47]. Jump height was recorded using the Perform Better^®^ Just Jump System. The Just Jump System has been validated against a 3-camera motion analysis system for estimating vertical jump height in a sample of males and females between the ages of 18–25 [48]. Participants were instructed to stand on the mat with their feet shoulder-width apart, place their hands on their hips, lower into the jump position (knees at approximately a 90° angle), hold for 2 seconds, explode upwards as high as possible, and land back on the mat. Participants performed three squat jumps separated by a 60 second recovery period. The highest jump was used to estimate muscle power.

Dynamic balance was measured using the Y-Balance Test. Briefly, after no more than four practice attempts, participants started by balancing on their left leg and then reached forward as far as they could and touched down. The distance was recorded, and the process was repeated two more times. The same process was then followed when balancing on the right leg. This was performed three times in each direction, alternating between balancing on the left and right feet. All six reaches per direction (left then right) were performed before moving to another direction.

Muscular endurance of the dominant knee extensors and flexors was assessed using a Humac^®^ NORM isokinetic dynamometer system (Computer Sports Medicine, Inc., Stoughton, MA, USA). Prior to testing, participants performed a 5-minute walking warmup. The participants were seated and secured to the device using straps across the trunk and thighs. The positioning of the seat was adjusted to the comfort level of the participant, so long as the approximate axis of the knee (through the lateral femoral epicondyle) was aligned with the dynamometer’s mechanical axis, and recorded so the same settings were used following the intervention. Range of motion was then prescribed on an individual basis (0° corresponds to full knee extension). Prior to testing, participants performed five repetitions at 120°/s as a familiarization. Upon completion of the familiarization, participants were given a two-minute recovery period before testing commenced. The testing protocol consisted of 30 reciprocal maximal contractions of the knee extensors and flexors performed at 180°/s, as previously described [49]. Total work, mean power per repetition, and peak torque were recorded.

### Statistical analysis

To test for normality within the sample, Shapiro-Wilk test was performed and confirmed with a visual examination of the data. General characteristics of the sample are presented as mean ±  standard deviation (SD) for continuous variables and n (%) for categorical variables, along with their effect sizes. Effect sizes were calculated using Hedges *g* formula, which is calculated by dividing the difference between the means by the pooled weighted standard deviation. Hedges *g* was selected as a measure of effect size as it incorporates a correction for small sample sizes to reduce risk of bias and provide a more accurate estimate of effect [50]. Effect sizes were interpreted as follows: Small effect: 0.00 ≤  *g* ≤  0.50, Medium effect: 0.50 ≤  *g* ≤  0.80, Large effect: *g* ≥  0.80. Differences in baseline and post-intervention values, grouped by sex, were analyzed using paired sample t-tests. For baseline and general characteristics, independent t-tests were performed to assess differences between groups. A repeated measures ANOVA was performed to determine whether there were significant main effects for an interaction between time and sex with changes in primary and exploratory outcomes. Data management and statistical analyses were performed using SPSS (IBM Corp. Released 2023. IBM SPSS Statistics for Windows, Version 29.0, Armonk, NY: IBM Corp). A *p* ≤  0.05 was considered significant.

## Results

### Descriptive characteristics

The total number of participants that completed the study was 38: 19 males and 19 females. An overview of the baseline descriptive characteristics is outlined in Table 1. Briefly, the mean age of males was 24.2 ±  2.76 years, and was 22.7 ±  3.37 years (*p* = 0.135) for females. Approximately 66% of the total sample were White (males =  68.4%, females =  60%). There were no statistically significant differences at baseline in weight, BMI, waist circumference, or steps per day (all *p-*values > 0.05). High-density lipoprotein levels were significantly higher in females compared to males (*p* = 0.002).

**Table 1:** General characteristics of the study sample.

	Total (n = 38)	Male (n = 19)	Female (n = 19)
Age (years)	23.5 ± 3.13	24.2 ± 2.76	22.7 ± 3.37
*Ethnicity*			
East Asian n (%)	6 (15.8)	0 (0)	6 (31.8)
Black n (%)	2 (5.3)	1 (5.3)	1 (5.3)
Indian n (%)	4 (10.5)	4 (21.1)	0 (0)
Indigenous n (%)	1 (2.6)	1 (5.3)	0 (0)
White n (%)	25 (65.8)	13 (68.4)	12 (60.0)
*Activity levels*			
Physical Activity (steps/day)	6496 ± 2198	6374 ± 2179	6619 ± 2269
*Metabolic profile*			
Cholesterol (mmol/L)	4.33 ± 0.87	4.05 ± 0.82	4.54 ± 0.86
HDL (mmol/L)	1.44 ± 0.39	1.27 ± 0.33	1.61 ± 0.37^*^
LDL (mmol/L)	2.28 ± 0.74	2.11 ± 0.75	2.41 ± 0.73
Triglycerides (mmol/L)	1.36 ± 0.98	1.60 ± 1.32	1.15 ± 0.46
Glucose (mmol/L)	5.24 ± 1.68	5.60 ± 2.36	4.89 ± 0.41

Variables are presented as means ±  standard deviation. HDL, high-density lipoproteins; LDL, low-density lipoproteins.

* Represents significant difference between groups using an independent sample t-test. Alpha level at 0.05.

### Changes in body composition

Table 2 describes the impact of six-week BFRT on body composition in males and females. Baseline fat mass was not significantly different between males and females (*p* = 0.303), but baseline lean mass was significantly greater in the male group (*p* < 0.001). Males significantly increased weight, BMI, and waist circumference (*p* = 0.006, 0.046, 0.025), whereas females saw no significant changes in these anthropometric measures. A significant interaction was observed for waist circumference (p = 0.022) and lower limb lean mass (p = 0.028). No significant changes were observed in males’ or females’ body fat percentage or fat mass.

**Table 2:** Body composition of male and female groups.

	Males (n = 19)				Females (n = 19)			
	Pre	Post	Effect Size (*g*)	*p*	Pre	Post	Effect Size (*g*)	*p*
*Anthropometrics*								
Weight (kg)	86.4 ± 21.5	87.9 ± 22.5	0.07	.006	72.6 ± 20.9	73.2 ± 20.2	0.03	0.161
Body mass index (kg/m^2^)	27.2 ± 6.83	27.7 ± 7.18	0.07	.006	26.8 ± 6.40	27.0 ± 6.15	0.03	0.138
Waist circumference (cm)	94.5 ± 16.0	96.4 ± 16.1	0.12	.025	88.0 ± 12.9	87.9 ± 11.0	−0.01	0.304^*^
*Body composition*								
Body fat (%)	26.8 ± 8.06	26.7 ± 8.04	−0.01	.521	38.0 ± 5.86	37.5 ± 5.80	−0.08	0.055
Fat mass (kg)	23.8 ± 12.2	24.1 ± 12.4	0.02	.200	27.8 ± 11.6	27.7 ± 11.4	−0.01	0.426
Upper limb lean mass (kg)	6.46 ± 1.07	6.59 ± 1.10	0.12	.051	3.61 ± 0.83	3.66 ± 0.82	0.06	0.056
Lower limb lean mass (kg)	19.8 ± 2.73	20.4 ± 3.20	0.20	.004	13.6 ± 3.13	13.7 ± 3.09	0.03	0.153^*^
Trunk lean mass (kg)	28.1 ± 6.15	28.4 ± 6.24	0.05	.146	20.6 ± 4.94	21.1 ± 4.55	0.10	0.050

Variables are presented as means ±  standard deviation. VAT, visceral adipose tissue.

* Represents significant interaction effect (time x sex) using a repeated measures analysis of variances. Alpha level at 0.05. Small effect: 0.00 ≤  *g* ≤  0.50, Medium effect: 0.50 ≤  *g* ≤  0.80, Large effect: *g* ≥  0.80.

**Fig 3:**
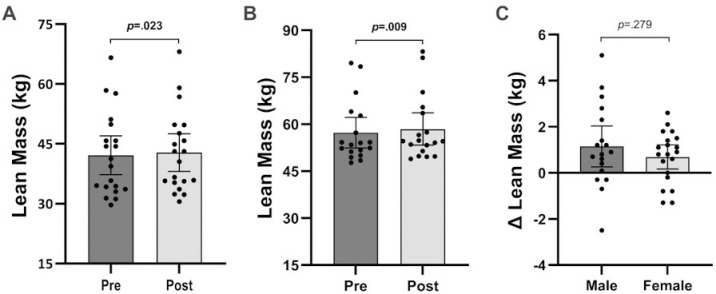
**A.** Lean mass of females at baseline and post-testing. **B.** Lean mass of males at baseline and post-testing. **C.** Absolute change of lean mass for males and females. Data are presented as mean and 95% confidence intervals with *p*-values.

**Fig 4:**
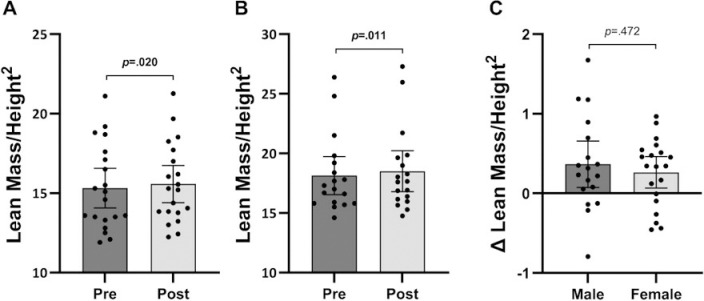
**A.** Relative lean mass of females at baselines and post-testing. **B.** Relative lean mass of males at baseline and post-testing. **C.** Absolute change of relative lean mass for males and females. Data are presented as mean and 95% confidence intervals with *p*-values.

Males (*p* = 0.009, *g* = 0.11) and females (*p* = 0.023, *g* = 0.08) significantly increased lean mass following six-week BFRT (Figs 3A and 3B). Similar results were observed for the impact of BFRT on relative lean mass in males (*p* = 0.011, *g* = 0.09) and females (*p* = 0.020, *g* = 0.08; Figs 4A and 4B). Repeated measures ANOVA revealed a time effect for lean body mass (*p* < 0.001) but no interaction between time and sex (*p* = 0.279, Fig 3C). Similar results were observed for relative lean mass, with a time effect (*p* < 0.001) and no interaction between time and sex (*p* = 0.472, Fig 4C).

### Changes in strength and performance outcomes

**Fig 5:**
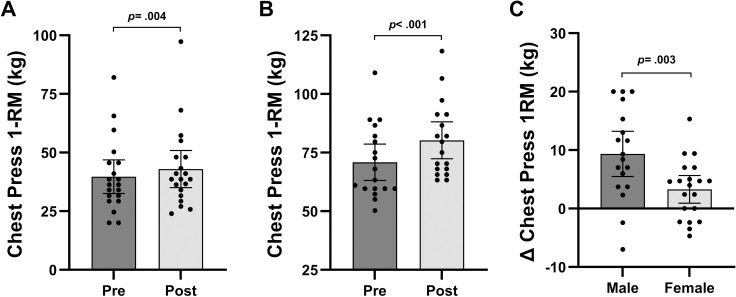
**A.** 1-RM for chest press of females at baseline and post-testing. **B.** 1-RM for chest press of males at baseline and post-testing. **C.** Absolute change of 1-RM for chest press from baseline to post-testing of males and females. Data are presented as mean and 95% confidence intervals with *p*-values.

**Fig 6:**
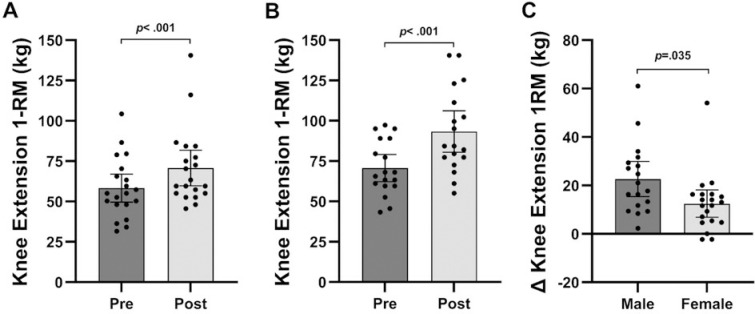
**A.** 1-RM for knee extension of females at baseline and post-testing. **B.** 1-RM for knee extension of males at baseline and post-testing. **C.** Absolute change of 1-RM for knee extension from baseline to post-testing of males and females. Data are presented as mean and 95% confidence intervals with *p*-values.

Table 3 shows changes in performance outcomes, while Figs 5A–C and 6A–C show changes in 1-RM from baseline to post-testing for chest press and knee extension, respectively, for males and females. Exception for females’ leg press (*p* = 0.110), males and females significantly improved knee extension (male: *p* < 0.001.; female: *p* < 0.001), knee flexion (*p* < 0.001; *p* < 0.001), chest press (*p* < 0.001; *p* = 0.004), and seated row (*p* = 0.003; *p* = 0.004) following 6-week BFRT. Repeated measures ANOVA revealed a time effect for leg press (time: *p* = 0.002), knee extension (*p* < 0.001), knee flexion (*p* < 0.001), chest press (*p* < 0.001), and seated row (*p* < 0.001), while an interaction effect was only observed for knee extension (*p* = 0.035), knee flexion (p = 0.043), chest press (*p* = 0.003), and seated row (*p* = 0.038).

**Table 3:** Performance outcomes of male and female groups.

	Males (n = 19)				Females (n = 19)			
	Pre	Post	Effect size (*g*)	p	Pre	Post	Effect size (*g*)	p
*Vertical jump*								
Jump peak power (Nm)	4296.7 ± 674.0	4545.7 ± 749.4	0.46	<0.001	2897.2 ± 815.6	3012.7 ± 742.6	0.15	0.002^*^
*Dynamic balance*								
Y-Balance right Score	84.9 ± 7.64	89.6 ± 9.06	0.55	0.002	83.5 ± 8.17	86.0 ± 6.34	0.33	0.159
Y-balance left Score	84.8 ± 8.10	89.5 ± 8.46	0.56	0.002	84.0 ± 7.33	86.2 ± 7.48	0.29	0.180
*Isokinetic dynamometry*								
KF peak torque (Nm)	61.1 ± 21.0	71.6 ± 27.2	0.42	0.005	41.2 ± 12.0	47.2 ± 14.6	0.44	0.010
KE peak torque (Nm)	150.5 ± 29.2	159.5 ± 34.0	0.28	0.055	90.1 ± 21.2	96.5 ± 18.9	0.31	0.033
KF total work (Nm)	1342.8 ± 546.6	1649.3 ± 645.0	0.50	0.008	1005.9 ± 417.4	1166.2 ± 391.0	0.39	0.032
KE total work (Nm)	3695.3 ± 798.0	4174.8 ± 697.0	0.63	0.016	2281.3 ± 642.0	2541 ± 539.8	0.43	<0.001
KF mean power (W)	99.9 ± 37.1	116.6 ± 40.1	0.42	0.010	64.1 ± 23.4	77.6 ± 23.0	0.57	0.002
KE mean power (W)	232.4 ± 53.4	254.8 ± 62.2	0.38	0.031	135.2 ± 41.7	153.2 ± 31.3	0.48	0.003
Total work combined (W)	5038.1 ± 1139.6	5824.1 ± 1183.6	0.66	0.006	3287.3 ± 969.0	3707.7 ± 837.5	0.45	0.002

Variables are presented as means ±  standard deviation. KF, knee flexors; KE, knee extensors.

* Represents significant interaction effect (time x sex) using a repeated measures analysis of variance. Alpha level at 0.05. Small effect: 0.00 ≤  *g* ≤  0.50, Medium effect: 0.50 ≤  *g* ≤  0.80, Large effect: *g* ≥  0.80.

Both males and females increased peak power produced during the vertical jump (male: *p* < 0.001; female: *p* = 0.002), and males increased jump peak power significantly more than females as an interaction between time and sex was observed (*p* = 0.039). Both males and females significantly improved their mean power, peak torque, and total work for their dominant knee flexors (all *p*-values < 0.05). Females improved mean power, peak torque, and total work for their dominant knee extensors (all *p*-values < 0.05). Males improved mean power (*p* = 0.031) and total work (*p* = 0.016), but not peak torque (*p* = 0.055) for their dominant knee extensors. There were no significant differences in the improvements between males and females as indicated by the interactions between time and sex (all *p*-values > 0.05).

## Discussion

The main objective of this study was to investigate sex-based differences and to compare the impact of 6-week BFRT on lean body mass, muscle strength, and performance outcomes between males and females. The principal finding of our study suggested a lack of sex differences for change in lean body mass following BFRT and showed a sex difference for muscle strength and performance outcomes (peak power). These results are pertinent as they fill the gap in the literature about the impact of BFRT and sex-based differences in adaptation for lean body mass, muscle strength, and performance outcomes. This is of great interest as it might help guide future research on sex differences in specific populations, such as in sport-specific athletes and injury rehabilitation.

Six weeks of BFRT resulted in a significant increase in lean body mass and relative lean body mass in both males and females. Furthermore, there was no difference between males and females for this change in absolute or relative lean body mass. Our findings corroborate previous findings of studies showing that BFRT induces muscle hypertrophy [10,12,15,37,51–54]. However, our results are surprising considering physiological sex differences have been documented in the literature with respect to muscle fiber types [18,20]. For example, it was reported that males have a lower proportion of Type I muscle fibers and a greater proportion of Type II muscle fibers [18,20]. Secondly, it has been shown that females undergo less metabolic stress during high-intensity exercise than males, which could translate to less of an adaptive stimulus [18].

Together, these sex differences were our rationale for hypothesizing a sex-specific difference in lean body mass adaptation. Nevertheless, the lack of sex differences in relative lean body mass from our study aligns with other studies that reported that both sexes appear to adapt to resistance training to a similar extent [12,55]. Therefore, the findings of this present study are novel as they add to the body of literature by directly comparing males and females and show an absence of any hypertrophic sex-based differences as males and females underwent similar whole-body muscle hypertrophy following only six weeks BFRT.

Our results also showed a significant increase in muscle strength, which is supported by previous systematic reviews [16,17]. However, we showed a sex-specific difference for muscle strength with males increasing knee extension, knee flexion, chest press, and seated row 1-RM to a greater extent than females. In a systematic review of the impact of BFRT on strength, Gear et al. (2022) showed that female studies had a greater effect size for muscle strength than the mixed or the male-only studies [17]. This difference could be explained by the very few female-only studies included (only two), which could impact the effect size. In support of our findings, sex differences in upper-body muscle strength have been shown following traditional resistance training, even when accounting for differences in lean body mass [56]. This sex-difference in upper-body strength could be due to differences in lean mass distribution between males and females, where males have been shown to have greater lean mass in the upper limbs and upper trunk [20,57,58]. Altogether, our findings continue to add to the current body of literature by directly comparing males and females and showing that only six weeks of BFRT is sufficient for increasing muscular strength in both sexes, but males may increase strength to a greater extent.

While some studies have found BFRT to improve jump performance [59–61], others have not [62,63]. Our findings compared males and females and showed that jump power is significantly increased after six weeks of whole-body BFRT in physically inactive young adults, and that males improved to a greater extent. Males may have increased their jump performance to a greater extent than the females due to them seeing a greater increase in lower-limb lean mass. Furthermore, as previously demonstrated, males tend to have a greater area of muscle occupied by Type II muscle fibers than females, which would contribute more to an explosive movement such as a vertical jump [20]. In fact, it has been shown that following six weeks of low-load BFRT in untrained men and women, males increased the cross-sectional area of their Type II muscle fibers to a greater extent than females [12]. Therefore, we demonstrated sex-based differences in power produced during a vertical jump following six weeks of BFRT that could be explained by a greater development of Type II muscle fibers following BFRT in men than in women.

Following 6-week BFRT, we demonstrated that males and females similarly improved their muscular endurance performance. A meta-analysis suggests that low-load BFRT is effective at increasing knee extensor and knee flexor peak torque, which is in line with our findings [16]. Previous findings have also shown that BFRT performed to failure is capable of improving muscle power during a similar high-intensity isokinetic fatigue test in healthy males, which our findings corroborate [13]. Interestingly, Korkmaz et al. (2020) observed significant improvements in the dominant knee extensors concentric isokinetic peak torque in the BFRT group over the traditional resistance exercise group (study only included males), whereas that is the only metric that was not improved in our male sample [52]. This discrepancy could be due differences in samples, where the sample in the study by Korkmaz et al. (2020) were trained male soccer athletes and ours were young adults who did not meet the physical activity guidelines [52].

Interestingly, other lower-body performances in our study were either improved in males and not in females, or improved to a greater extent in males, but not muscular endurance. The difference in lower-limb lean mass seen in the males may have been offset by females potentially having greater fatigue-resistance to repeated knee extensions, as highlighted by Labarbera et al. (2013) [64]. The greater resistance to fatigue in females could be explained by the greater proportional area of Type I muscle fibers which translates to a greater oxidative capacity than in males, and in turn less contractile dysfunction during high-intensity exercise [18]. Our results continue to build on previous findings by highlighting how BFRT impacts muscle strength and muscle endurance, and by documenting sex-specific differences in performance outcomes in young adults not meeting the physical activity guidelines.

### Strengths and limitations

Our work has some limitations that need to be highlighted. First, our intervention had a short duration of only six weeks, and it is possible that some sex-specific differences in outcomes could have occurred over longer durations of BFRT. Second, we did not include a control condition in our study which limited the conclusion drawn from this study. Finally, although we asked participants to maintain their current lifestyle, including diet, we did not control diet every week for the duration of the study. While it has been recommended to include females in exercise science research with 1) pre-defined and standardized inclusion criteria and 2) to adapt the experimental design with consideration of menstrual cycle or hormonal contraceptive factors [65], recent evidence suggests that the impact of the menstrual cycle or hormonal contraceptives is nonexistent [66,67]. Although we did not control for menstrual cycle, current evidence suggests that it is unnecessary, and the emphasis should be to include females in exercise physiology research that is adequately powered to address their underrepresentation and to answer the research question [67]. However, our work is strengthened by the compliance of participants to the study – every participant completed 100% of the exercise sessions. Also, exercise sessions were supervised in very small groups by research staff, allowing for a tightly controlled environment. Furthermore, our work is strengthened by our sufficiently powered sample to directly compare males and females, allowing us to draw insights on potential sex differences in lean mass, muscle strength, and performance outcomes following BFRT.

## Conclusion

In summary, our findings suggest that following 6-week whole-body BFRT, males and females significantly increase lean body mass and muscle endurance without sex differences. Moreover, BFRT significantly increased muscle strength and power for males and females. However, males increase muscle strength and power to a greater extent than females. Altogether, our research adds novelty to the current body of literature by documenting sex differences following BFRT. Future studies should continue to investigate sex differences following BFRT, but over longer durations, with different populations, with a focus on the underlying physiological mechanisms underpinning these adaptations, and how these differences may influence health and performance.

## Supporting information

S1 File Study protocol. (DOC)

S2 File CONSORT checklist. (DOCX)

## Abbreviations

ANOVA: analysis of variance
BFRT: blood flow restriction training
BMI: body mass index
DXA: dual-energy x-ray absorptiometry
1-RM: 1-repetition maximum
