# Supplementary material for: Impact of exercise with blood flow restriction on muscle hypertrophy and performance outcomes in men and women
Source: PLoS One. 2025 Jan 28;20(1):e0301164. doi: 10.1371/journal.pone.0301164 (PMC11774385; doi:10.1371/journal.pone.0301164)
Supplement: S2 File — (DOCX) [file pone.0301164.s002.docx]

## **Research Protocol**

## **Background:** Resistance training has been described as a “medicine,” as it has the ability to prevent several chronic conditions and contribute to maintaining good health (1,2). Likewise, it is generally understood that resistance training is beneficial for healthy, older, and injured populations. Specifically, it has been shown that resistance training can aid in the development of fitness and maintainence of health, which in turn protects against a number of chronic conditions such as type 2 diabetes mellitus, cardiovascular disease, cancer, and others (2). One potential reason for the benefits of resistance training is the change in body composition that occurs. Although resistance training may reduce fat mass, generally it leads to an addition of skeletal muscle mass (3–5). This addition of muscle is known as hypertrophy. It was well established that muscle hypertrophy occurs following moderate to heavy-load resistance training as well as resistance training to volitional failure using a lighter load (6,7). More recently a novel form of resistance training has emerged as an alternative to heavy-load resistance training. This type of resistance training is known as blood flow restriction training (BFRT) and combines very light loads with the use of a pneumatic cuff placed around the most proximal region of the exercising limb to partially restrict the blood flow (8).

Briefly, BFRT (also known as KAATSU Training for the company that developed it) originated in Japan and has been developed over time since the mid-1900s. Since the 1990s it has undergone rigorous academic research to determine its efficacy as a low-load resistance training alternative. BFRT has been used in a wide range of populations; including elite and high-performance athletes, rehabiliating populations, and elderly adults (9). In essence, BFRT allows for light loads to be used during resistance training by altering the blood flow into the exercising muscles and occluding the blood flow from leaving the muscles, inducing a high degree of metabolic stress which in turn is believed to stimulate muscle adaptation (8,10). Although BFRT is an effective low-load alternative to traditional heavy-load resistance training, there are potential risks with exercise of any kind and BFRT must be undertaken with care to ensure the safety of users. A national survery of KAATSU users in Japan found an extremely low incidence rate for any injuries while doing BFRT (11). Likewise, a number of best-practice guidelines and evidence-based reviews have been published to ensure safe use of BFRT (8,12,13).

The vast majority of studies investigating BFRT include only college-aged males (i.e., between the ages of 18-25) (14). In fact, a call to action in a 2018 review highlights that in studies of chronic and acute (acute meaning one bout of exercise and chronic implying repeated bouts of exercise) BFRT, only 29% and 17%, respectively, of all participants are females (15). This fact necessitates an urgent response. There is no reason for the exclusion of women in BFRT research.

Due to the limited evidence for BFRT with respect to women, the current project aims to provide insight as to how males and females may differ in response to BFRT following six weeks of whole-body resistance training combined with blood flow restriction. Therefore, the objective of this study is to investigate the impact of 6-week, whole-body BFRT on lean body mass, strength, and functional outcomes in males and females with the goal of adding to the growing body of literature, guiding future research on sex differences, and allowing for more specific prescription of this exercise in female populations.

## **Rationale:** There is a substantial gap in the literature surrounding how males and females may be impacted differently following BFRT. Although some studies exist examining sex differences following a BFRT intervention, these studies have investigated outcomes such as electromyography signal (16,17) , muscle fatigue based on torque and maximal voluntary contraction (17,18), metabolic responses (16), and post-exercise application of blood flow restriction (19). No study thus far has investigated whether muscle hypertrophy response following a chronic BFRT intervention differs between the sexes. However, Wells et al. (2019) did observe muscle hypertrophy following a four-week BFRT intervention in a female-only and reported that their findings were similar to previous results observed in males (20). However, no further study has supported or refuted this finding.

While increasing muscle mass might have previously been viewed as an athletic endeavour and weight loss viewed as a health endeavour, but it is now understood that fat mass and muscle mass play independent roles in long-term health and each serve their own purpose. By better understanding the effects of BFRT on lean body mass of males and females, better prescription of exercise can be utilized to maximize lean body mass increases.

## **Objective:** Determine whether any differences in lean body mass or strength are observed between males and females following a six-week whole-body resistance training program with blood flow restriction.

## **Methodology**

### **Study Design:** The current project is a parallel control experimental study comparing men and women following a 6-week resistance training intervention in conjunction with partial blood-flow restriction. (Clinical Trial #: NCT05615831). Briefly, participants will undergo baseline testing separated into two visits within a one-week span, presenting at the Cardiometabolic Exercise and Lifestyle Laboratory at the University of New Brunswick for each visit. Participants will begin six weeks of BFRT within one week of their last baseline testing visit. Following the intervention, participants will undergo follow-up testing at least two days (21), but no more than one week (22), following their last exercise session. All participants will be required to provide written and informed consent prior to participation. The project is being reviewed by the University of New Brunswick Research Ethics Board (REB 2021-124).

###

### **Sample Size:** A power calculation was performed using G-power software (version 3.1.9.4, Germany) to determine the appropriate sample size for statistical significance. Based on an alpha of 0.05, a power of 0.8, and an effect size of 0.4, we determined the required total sample size to be eight participants for a repeated measures analysis of variance (ANOVA). However, we anticipate a dropout percentage of 13% as reported by Høgsholt et al. (2022) (23). Furthermore, it has been suggested that in order to detect interaction effects between sexes, as well as main effects, the sample size needs to be four-times the size (24). To account for dropout rate, and to ensure adequate ability to detect interaction and main effects, we are attempting to recruit approximately 20 per group for a total of 40 participants.

### **Participation**

#### **Inclusion Criteria:** Participants will be eligible for inclusion if they are between the ages of 19 and 30 years. In addition, participants need to be physically inactive, but otherwise healthy. Physical inactivity is defined as not meeting the World Health Organization’s 2020 physical activity guidelines: 150 minutes of moderate-vigorous physical activity and two muscle-strengthening activities per week (25). Physical activity levels will be estimated through questionnaires and using Fitbit Charge 3 activity trackers. It was shown in a 2018 systematic review that Fitbit activity trackers provide accurate measures of steps in adults with no mobility limitations (26). Using a pre-determined threshold (10,000 steps/day) as the minimum number of steps required to reach moderate intensity physical activity, anybody who averages under 10,000 steps/day over a 4–7-day window and does not perform muscle strengthening activities twice per week will be considered physically inactive (27).

#### **Exclusion Criteria:** Exclusion criteria includes: 1) aged outside prearranged threshold (19 – 30 years), 2) the presence of cardiovascular disease such as coronary heart disease, uncontrolled hypertension, peripheral vascular disease, venous thromboembolism, other blood clotting disorders, or hemophilia, 3) surgery, bone fracture, or a skin graft within the last three months, 4) pregnancy, and 5) meeting or exceeding physical activity guidelines.

#### **Recruitment:** Recruitment will be performed between May 2022 and July 2023 through the distribution of promotional flyers, University of New Brunswick’s newsletter, and social media advertisements through Facebook and Instagram.

### **Exposure Variable – Blood Flow Restriction Training:** Participants will undertake 6 weeks of whole-body resistance training in conjunction with blood flow restriction to their exercising limbs. The intervention consists of three supervised exercise sessions per week consisting of five different exercises: knee flexion (hamstring curl), knee extension, leg press, chest press, and seated row. The exercise load will be individualized to 30% of each participant’s 1-RM for each exercise. Participants are required to complete 75 total repetitions broken into four sets for each exercise. The sets are broken up in the following manner: set 1: 30 repetitions; set 2: 15 repetitions; set 3: 15 repetitions; set 4: 15 repetitions, as this protocol has previously been used in blood flow restriction research to induce muscle hypertrophy in a variety of populations (28,29) and has been suggested by multiple reviews (13,30). At week 4, participants will have their 1-RM reassessed to adjust the 30% 1-RM exercising loads. This will occur during the first exercise session of Week 4. As such, following the 1-RM reassessment, participants will perform two sets per exercise (30 reps and 15 reps) using the newly adjusted 30% 1-RM weight, before returning to the original rep scheme for their next session.

Blood flow restriction cuffs will be placed at the most proximal portion of the exercising limb (just above biceps brachii on the arm and near the inguinal crease on the thigh), which has previously been used in BFRT research (8,13,28,29,31–34). Blood flow restriction was achieved using the KAATSU C3 device (KAATSU Global, Inc., Huntington Beach, CA, USA). The KAATSU arm and leg cuffs are 5 cm wide, respectively, and are single-bladder cuffs. Cuffs will be inflated to 60% of each individual’s total limb occlusion pressure as this has been shown to be a safe and effective pressure to induce muscular adaptations (35), and is within the recommended pressure range for BFRT (8,13). Each participant’s total limb occlusion pressure (LOP) will be estimated using equations developed by Loenneke et al. (2015) listed here (31):

1. $Leg arterial occlusion \left( mmHg \right)=5.893\left( thigh circumference \right)+0.734(diastolic blood pressure)+0.912(systolic blood pressure)-220.046$
2. $Arm arterial occlusion \left( mmHg \right)=0.514\left( systolic blood pressure \right)+0.339\left( diastolic blood pressure \right)+1.461\left( arm circumference \right)+17.236$

Although these equations have modest R-squared values of 0.61 and 0.49, respectively, they have been successfully utilized in a BFRT intervention of trained athletes leading to significant improvements in muscle hypertrophy and functional outcomes with no reported side effects (36). Furthermore, although LOP will be estimated, a study comparing the impact of various cuff pressures showed similar effects on muscle size, torque, strength, and endurance between 40% and 90% pressures (37). These findings suggest that greater pressures are not necessarily more effective at inducing adaptations and that small pressure fluctuations will not necessarily serve as greater or lesser stimuli during training. Cuffs will remain inflated during the rest in between sets of each exercise but will be deflated for the rest period between exercises (28,38–41). The set rest is 60 seconds, and the rest between exercises is four minutes.

### **Primary Outcome Measure:** Lean body mass will be estimated using duel-energy x-ray absorptiometry (DXA) prior to the 6-week BFRT intervention, and again following the intervention. Body composition will be estimated using a Hologic Horizon^®^ DXA System (Hologic Canada ULC, Mississauga, ON, Canada). Lean body mass constitutes that which is not fat mass nor bone mineral mass (42). Participants will present to the laboratory following a 12-hr fast and are asked to refrain from exercise for a 24-hr period prior to testing. Participants will be instructed to wear loose-fitting clothing with no metal (buckles, zippers, buttons, etc.) and then instructed to lie supine on the scanner’s table and remain still for the duration of the scan. Arms will be placed at the participants’ sides with palms facing medially and thumbs pointed upwards. For individuals too large for the width of the table, they will be positioned with one arm outside of the scan area and results of the scanned arm duplicated. The coefficient of variation in our lab for lean mass is 0.6% and for body fat percentage is 0.7%. This was performed on 33 people (males, n=10) with a mean age of 23.4 years and a mean body mass index (BMI) of 25.6.

###

### **Exploratory Variables:** Anthropometric measurements, muscular power and endurance, and strength will be measured for exploratory purposes and sample description. Participants’ height and weight will be measured to the nearest 0.5 cm and 0.1 kg, respectively, according to the CSEP protocol (43). Weight will be measured using a calibrated column scale (SECA^®^ model #213). Height will be measured using a standardized stadiometer. With no shoes, feet together, and arms at their side, height is taken following an inhalation. Hip and waist circumference are measured using an anthropometric tape measure and recorded to the nearest 0.5 cm. For hip and waist measurements, participants stand with their feet shoulder-width apart and their arms folded across their chest. Waist circumference is measured at the upper lateral border of the iliac crest following a normal exhalation, hip circumference is measured around the widest portion of the buttocks after a normal exhalation (43).

Strength will be assessed by 1-RM for the five exercises used during the intervention. 1-RM will be measured during the second baseline testing visit, at the midpoint of the study during the first exercise session of week four, and again during the second testing visit in the follow-up testing. Each participant’s 1-RM will be determined using the following protocol: one set of 6-10 repetitions, followed by one set of 3-5 repetitions, followed by small incremental increases for one repetition until a failure is achieved within seven attempts. If no failure is achieved within seven attempts, the 1-RM for that exercise will be redone prior to their first exercise session.

Muscular power will be estimated using the squat jump equation derived by Sayers et al. (1999) and is as follows (44):

1. $Peak Power \left( W \right)=60.7 x \left( jump height \left[ cm \right] \right)+45.3 x \left( body mass \left[ kg \right] \right)-2055$

This equation was chosen as it has been shown to be more accurate than previously used power estimation equations and was developed from a large and diverse population which enhances our external validity. Jump height will be recorded using the Perform Better^®^ Just Jump System. The Just Jump System has been validated against a 3-camera motion analysis system for estimating vertical jump height in a sample of males and females between the ages of 18-25 (45). Participants will be instructed to stand on the mat with their feet shoulder-width apart, place their hands on their hips, lower into the jump position (knees at approximately a 90° angle), hold for 2 seconds, explode upwards as high as possible, and land back on the mat. Participants will perform three squat jumps separated by a 60-second recovery period. The highest jump will be used to estimate muscle power.

Dynamic balance will be measured using the Y-Balance Test. Briefly, after no more than four practice attempts, participants will start by balancing on their left leg and then reaching forward as far as they can and touch down. The distance will be recorded, and the process repeated two more times. The same process will then be followed when balancing on the right leg. This will be performed three times in each direction, alternating between balancing on the left and right feet. All six reaches per direction (left then right) will be performed before moving to another direction.

Muscular endurance of the dominant knee extensors and flexors will be assessed using a Humac^®^ NORM isokinetic dynamometer system (Computer Sports Medicine, Inc., Stoughton, MA, USA). Prior to testing, participants will perform a 5-minute walking warmup. The participants will be seated and secured to the device using straps across the trunk and thighs. The positioning of the seat will be adjusted to the comfort level of the participant, so long as the approximate axis of the knee (through the lateral femoral epicondyle) is aligned with the dynamometer’s mechanical axis, and recorded so the same settings can be used following the intervention. Range of motion will then be prescribed on an individual basis (0° corresponds to full knee extension). Prior to testing, participants will perform five repetitions at 120°/s as a familiarization. Upon completion of the familiarization, participants will be given a two-minute recovery period before testing commences. The testing protocol consists of 30 reciprocal maximal contractions of the knee extensors and flexors performed at 180°/s, as previously described (46). Total work, average power per repetition, and peak torque will be recorded.

### **Statistical Analysis:** To test for normality within the sample, Shapiro-Wilk test will be performed and confirmed with a visual examination of the data. General characteristics of the sample will be presented as mean ± SD for continuous variables and n (%) for categorical variables, along with their effect sizes. Effect sizes will be calculated using Hedges *g* formula, which is calculated by dividing the difference between the means by the pooled weighted standard deviation. Differences in baseline and post-intervention values, stratified by sex, will be analyzed using paired sample t-tests. A repeated measures ANOVA will be performed to determine whether there was a significant interaction effect between time and sex with changes in primary and exploratory outcomes. Data management and statistical analyses will be performed using SPSS version 29. A *p* ≤ 0.05 will be considered significant.

**References**

1. Westcott WL. Resistance Training is Medicine: Effects of Strength Training on Health. Curr Sports Med Rep [Internet]. 2012;11(4). Available from: https://journals.lww.com/acsm-csmr/Fulltext/2012/07000/Resistance_Training_is_Medicine__Effects_of.13.aspx

2. Mcleod JC, Stokes T, Phillips SM. Resistance Exercise Training as a Primary Countermeasure to Age-Related Chronic Disease. Front Physiol [Internet]. 2019;10. Available from: https://www.frontiersin.org/article/10.3389/fphys.2019.00645

3. Wewege MA, Desai I, Honey C, Coorie B, Jones MD, Clifford BK, et al. The Effect of Resistance Training in Healthy Adults on Body Fat Percentage, Fat Mass and Visceral Fat: A Systematic Review and Meta-Analysis. Sports Med. 2022 Feb 1;52(2):287–300.

4. Benito PJ, Cupeiro R, Ramos-Campo DJ, Alcaraz PE, Rubio-Arias JÁ. A Systematic Review with Meta-Analysis of the Effect of Resistance Training on Whole-Body Muscle Growth in Healthy Adult Males. Int J Environ Res Public Health. 2020 Jan;17(4):1285.

5. Hagstrom AD, Marshall PW, Halaki M, Hackett DA. The Effect of Resistance Training in Women on Dynamic Strength and Muscular Hypertrophy: A Systematic Review with Meta-analysis. Sports Med. 2020 Jun 1;50(6):1075–93.

6. Schoenfeld BJ. The Mechanisms of Muscle Hypertrophy and Their Application to Resistance Training. J Strength Cond Res [Internet]. 2010;24(10). Available from: https://journals.lww.com/nsca-jscr/Fulltext/2010/10000/The_Mechanisms_of_Muscle_Hypertrophy_and_Their.40.aspx

7. Krzysztofik M, Wilk M, Wojdała G, Gołaś A. Maximizing Muscle Hypertrophy: A Systematic Review of Advanced Resistance Training Techniques and Methods. Int J Environ Res Public Health. 2019 Dec 4;16(24):4897.

8. Scott BR, Loenneke JP, Slattery KM, Dascombe BJ. Exercise with Blood Flow Restriction: An Updated Evidence-Based Approach for Enhanced Muscular Development. Sports Med. 2015 Mar 1;45(3):313–25.

9. Pignanelli C, Christiansen D, Burr JF. Blood flow restriction training and the high-performance athlete: science to application. J Appl Physiol. 2021 Apr 1;130(4):1163–70.

10. Manini TM, Clark BC. Blood Flow Restricted Exercise and Skeletal Muscle Health. Exerc Sport Sci Rev. 2009 Apr;37(2):78–85.

11. Nakajima T, Kurano M, Iida H, Takano H, Oonuma H, Morita T, et al. Use and safety of KAATSU training:Results of a national survey. Int J KAATSU Train Res. 2006;2(1):5–13.

12. Australian Sports Commission. Sport Australia. 2021 [cited 2022 Jan 20]. Blood flow restriction training guidelines. Available from: https://www.ais.gov.au/position_statements/best_practice_content/blood-flow-restriction-training-guidelines

13. Patterson SD, Hughes L, Warmington S, Burr J, Scott BR, Owens J, et al. Blood Flow Restriction Exercise: Considerations of Methodology, Application, and Safety. Front Physiol. 2019;10:533.

14. Freitas EDS, Karabulut M, Bemben MG. The Evolution of Blood Flow Restricted Exercise. Front Physiol [Internet]. 2021 [cited 2023 May 11];12. Available from: https://www.frontiersin.org/articles/10.3389/fphys.2021.747759

15. Counts BR, Rossow LM, Mattocks KT, Mouser JG, Jessee MB, Buckner SL, et al. Let’s talk about sex: where are the young females in blood flow restriction research? Clin Physiol Funct Imaging. 2018;38(1):1–3.

16. Ellefsen S, Hammarström D, Strand TA, Zacharoff E, Whist JE, Rauk I, et al. Blood flow-restricted strength training displays high functional and biological efficacy in women: a within-subject comparison with high-load strength training. Am J Physiol-Regul Integr Comp Physiol. 2015 Oct 1;309(7):R767–79.

17. Dankel SJ, Buckner SL, Jessee MB, Mattocks KT, Mouser JG, Counts BR, et al. Post-exercise blood flow restriction attenuates muscle hypertrophy. Eur J Appl Physiol. 2016 Oct 1;116(10):1955–63.

18. Freitas EDS, Galletti BRA, Koziol KJ, Miller RM, Heishman AD, Black CD, et al. The Acute Physiological Responses to Traditional vs. Practical Blood Flow Restriction Resistance Exercise in Untrained Men and Women. Front Physiol [Internet]. 2020;11. Available from: https://www.frontiersin.org/article/10.3389/fphys.2020.577224

19. Gil ALS, Neto GR, Sousa MSC, Dias I, Vianna J, Nunes RAM, et al. Effect of strength training with blood flow restriction on muscle power and submaximal strength in eumenorrheic women. Clin Physiol Funct Imaging. 2017 Mar 1;37(2):221–8.

20. Wells E, Eustace D, Gupton CS, Dedrick GS, Bunn J. Eccentric and blood flow restriction exercises in women induce hypertrophy. J Sports Med Phys Fitness. 2019 Dec;59(12):1968–74.

21. Thiebaud RS, Yasuda T, Loenneke JP, Abe T. Effects of low-intensity concentric and eccentric exercise combined with blood flow restriction on indices of exercise-induced muscle damage. Interv Med Appl Sci. 2013 Jun;5(2):53–9.

22. Davids CJ, Næss TC, Moen M, Cumming KT, Horwath O, Psilander N, et al. Acute cellular and molecular responses and chronic adaptations to low-load blood flow restriction and high-load resistance exercise in trained individuals. J Appl Physiol. 2021 Dec;131(6):1731–49.

23. Høgsholt M, Jørgensen S, Rolving N, Mechlenburg I, Tønning L, Bohn M. Exercise With Low-Loads and Concurrent Partial Blood Flow Restriction Combined With Patient Education in Females Suffering From Gluteal Tendinopathy: A Feasibility Study. Front Sports Act Living. 2022 Apr 1;4:881054.

24. Rich-Edwards JW, Kaiser UB, Chen GL, Manson JE, Goldstein JM. Sex and Gender Differences Research Design for Basic, Clinical, and Population Studies: Essentials for Investigators. Endocr Rev. 2018 Aug 1;39(4):424–39.

25. Bull F, Al-Ansari S, Biddle S, Borodulin K, Buman M, Cardon G, et al. World Health Organization 2020 guidelines on physical activity and sedentary behaviour. Br J Sports Med. 2020;54(24):1451–62.

26. Feehan LM, Geldman J, Sayre EC, Park C, Ezzat AM, Yoo JY, et al. Accuracy of Fitbit Devices: Systematic Review and Narrative Syntheses of Quantitative Data. JMIR MHealth UHealth. 2018 Aug 9;6(8):e10527.

27. Tudor-Locke C, Craig CL, Brown WJ, Clemes SA, De Cocker K, Giles-Corti B, et al. How many steps/day are enough? for adults. Int J Behav Nutr Phys Act. 2011 Jul 28;8(1):79.

28. Letieri RV, Furtado GE, Barros PMN, Farias MJA de, Antunez BF, Gomes BB, et al. Effect of 16-Week Blood Flow Restriction Exercise on Functional Fitness in Sarcopenic Women: A Randomized Controlled Trial. Int J Morphol. 2019;37(1):59–64.

29. Ozaki H, Yasuda T, Ogasawara R, Sakamaki-Sunaga M, Naito H, Abe T. Effects of high-intensity and blood flow-restricted low-intensity resistance training on carotid arterial compliance: role of blood pressure during training sessions. Eur J Appl Physiol. 2013 Jan 1;113(1):167–74.

30. Kelly MR, Cipriano KJ, Bane EM, Murtaugh BT. Blood Flow Restriction Training in Athletes. Curr Phys Med Rehabil Rep. 2020 Dec 1;8(4):329–41.

31. Loenneke JP, Allen KM, Mouser JG, Thiebaud RS, Kim D, Abe T, et al. Blood flow restriction in the upper and lower limbs is predicted by limb circumference and systolic blood pressure. Eur J Appl Physiol. 2015 Feb 1;115(2):397–405.

32. Behringer M, Behlau D, Montag JCK, McCourt ML, Mester J. Low-Intensity Sprint Training With Blood Flow Restriction Improves 100-m Dash. J Strength Cond Res [Internet]. 2017;31(9). Available from: https://journals.lww.com/nsca-jscr/Fulltext/2017/09000/Low_Intensity_Sprint_Training_With_Blood_Flow.15.aspx

33. Luebbers PE, Fry AC, Kriley LM, Butler MS. The Effects of a 7-Week Practical Blood Flow Restriction Program on Well-Trained Collegiate Athletes. J Strength Cond Res [Internet]. 2014;28(8). Available from: https://journals.lww.com/nsca-jscr/Fulltext/2014/08000/The_Effects_of_a_7_Week_Practical_Blood_Flow.23.aspx

34. Yokokawa Y, Hongo M, Urayama H, Nishimura T, Kai I. Effects of low-intensity resistance exercise with vascular occlusion on physical function in healthy elderly people. Biosci Trends. 2008 Jun;2(3):117–23.

35. Brandner CR, Clarkson MJ, Kidgell DJ, Warmington SA. Muscular Adaptations to Whole Body Blood Flow Restriction Training and Detraining. Front Physiol [Internet]. 2019;10. Available from: https://www.frontiersin.org/article/10.3389/fphys.2019.01099

36. Manimmanakorn A, Hamlin MJ, Ross JJ, Taylor R, Manimmanakorn N. Effects of low-load resistance training combined with blood flow restriction or hypoxia on muscle function and performance in netball athletes. J Sci Med Sport. 2013 Jul 1;16(4):337–42.

37. Counts BR, Dankel SJ, Barnett BE, Kim D, Mouser JG, Allen KM, et al. Influence of relative blood flow restriction pressure on muscle activation and muscle adaptation. Muscle Nerve. 2016;53(3):438–45.

38. Cook SB, LaRoche DP, Villa MR, Barile H, Manini TM. Blood flow restricted resistance training in older adults at risk of mobility limitations. Exp Gerontol. 2017 Dec;99:138–45.

39. Amani-Shalamzari S, Rajabi S, Rajabi H, Gahreman DE, Paton C, Bayati M, et al. Effects of Blood Flow Restriction and Exercise Intensity on Aerobic, Anaerobic, and Muscle Strength Adaptations in Physically Active Collegiate Women. Front Physiol [Internet]. 2019;10. Available from: https://www.frontiersin.org/article/10.3389/fphys.2019.00810

40. Takada S, Okita K, Suga T, Omokawa M, Morita N, Horiuchi M, et al. Blood Flow Restriction Exercise in Sprinters and Endurance Runners. Med Sci Sports Exerc [Internet]. 2012;44(3). Available from: https://journals.lww.com/acsm-msse/Fulltext/2012/03000/Blood_Flow_Restriction_Exercise_in_Sprinters_and.7.aspx

41. Harper SA, Roberts LM, Layne AS, Jaeger BC, Gardner AK, Sibille KT, et al. Blood-Flow Restriction Resistance Exercise for Older Adults with Knee Osteoarthritis: A Pilot Randomized Clinical Trial. J Clin Med. 2019 Feb 21;8(2):265.

42. Prado CMM, Heymsfield SB. Lean Tissue Imaging. J Parenter Enter Nutr. 2014;38(8):940–53.

43. Canadian Society for Exercise Physiology. CSEP-PATH: Physical Activity Training for Health. 3rd ed. Canadian Society for Exercise Physiology; 2021.

44. Sayers SP, Harackiewicz DV, Harman EA, Frykman PN, Rosenstein MT. Cross-validation of three jump power equations. Med Sci Sports Exerc [Internet]. 1999;31(4). Available from: https://journals.lww.com/acsm-msse/Fulltext/1999/04000/Cross_validation_of_three_jump_power_equations.13.aspx

45. Leard JS, Cirillo MA, Katsnelson E, Kimiatek DA, Miller TW, Trebincevic K, et al. Validity of two alternative systems for measuring vertical jump height. J Strength Cond Res. 2007 Nov;21(4):1296–9.

46. Bosquet L, Gouadec K, Berryman N, Duclos C, Gremeaux V, Croisier JL. The Total Work Measured During a High Intensity Isokinetic Fatigue Test Is Associated With Anaerobic Work Capacity. J Sports Sci Med. 2016 Feb 23;15(1):126–30.
